# The Co-Administration of Paclitaxel with Novel Pyridine and Benzofuran Derivatives that Inhibit Tubulin Polymerisation: A Promising Anticancer Strategy

**DOI:** 10.3390/pharmaceutics17020223

**Published:** 2025-02-09

**Authors:** Magdalena Perużyńska, Radosław Birger, Patrycja Kłos, Halina Kwiecień, Łukasz Struk, Jacek G. Sośnicki, Laurence Lafanechère, Marek Droździk

**Affiliations:** 1 Department of Experimental and Clinical Pharmacology, Pomeranian Medical University in Szczecin, Powstanców Wielkopolskich 72, 70-111 Szczecin, Poland; radoslaw.birger@pum.edu.pl (R.B.); marek.drozdzik@pum.edu.pl (M.D.); 2 Department of Biochemistry and Medical Chemistry, Pomeranian Medical University in Szczecin, Powstanców Wielkopolskich 72, 70-111 Szczecin, Poland; patrycja.klos@pum.edu.pl; 3 Faculty of Chemical Technology and Engineering, West Pomeranian University of Technology in Szczecin, Piastów 42, 71-065 Szczecin, Poland; halina.kwiecien@zut.edu.pl; 4 Department of Organic and Physical Chemistry, Faculty of Chemical Technology and Engineering, West Pomeranian University of Technology in Szczecin, Piastów 42, 71-065 Szczecin, Poland; lukasz.struk@zut.edu.pl (Ł.S.); jacek.sosnicki@zut.edu.pl (J.G.S.); 5 Institute for Advanced Biosciences, University Grenoble Alpes, CNRS UMR 5309, INSERM U1209, F-38700 Grenoble, France; laurence.lafanechere@univ-grenoble-alpes.fr

**Keywords:** anticancer agents, anti-tubulin agents, pyridines, benzofurans, HeLa cells, paclitaxel, synergism

## Abstract

**Background**: Paclitaxel (PTX), a crucial microtubule-stabilising agent in cancer treatment, is limited by its adverse effects and hydrophobic nature, which necessitate the use of toxic solvents. This study proposes a novel approach combining PTX with new microtubule-destabilising compounds at low, safe doses that are ineffective when used individually. **Objective:** The aim was to evaluate the therapeutic efficacy of combining PTX with previously described pyridine (S1, S22) and benzofuran derivatives (13b, 14), which have demonstrated promising anticancer properties by inhibiting microtubule polymerisation. **Methods:** The PrestoBlue assay was used to determine the optimal concentrations of each compound, enabling synergistic interactions with a low dose of PTX in HeLa cervical cancer cells. The combined effects of the compounds and PTX on apoptosis, cell cycle distribution, and mitotic spindle formation were then evaluated. **Results:** The results showed that compounds 13b (1 µM), 14 (0.1 µM), S1 (2 µM), and S22 (2 µM) enhanced the proapoptotic and antimitotic effects of 1 nM PTX, which was ineffective alone. Notably, live-cell imaging revealed that the concurrent use of S1 and PTX produced effects similar to those of a higher PTX concentration (5 nM). **Conclusions:** These findings suggest that these compounds enhance the anticancer efficacy of low-dose PTX, potentially paving the way for more effective and safer cancer therapies.

## 1. Introduction

Despite significant progress in cancer therapy through the use of monoclonal antibodies and protein kinase inhibitors, microtubule-targeting agents (MTAs), also referred to as ‘spindle poisons’, remain one of the most important classes of anticancer drugs. MTAs include compounds that stabilise (taxanes, epothilones, laulimalide) and destabilise (colchicine, Vinca alkaloids, combretastatins) microtubules. Because microtubule dynamics are central to proper cell division, both groups of compounds lead to the formation of abnormal division spindles, cell cycle arrest in the G2/M phase, activation of spindle assembly checkpoints (SACs), and consequently, apoptosis of cancer cells [1]. Paclitaxel (PTX), initially obtained from the bark of *Taxus brevifolia*, was the first clinically used taxane [2,3]. PTX acts as an antimitotic agent that prevents microtubules depolymerisation and maintains their stability. This action results in the disturbance of mitotic spindles, causing cells to halt their cycle at the G2/M phase and ultimately undergo apoptosis [4,5]. The introduction of PTX and its semi-synthetic derivatives (docetaxel and cabazitaxel) revolutionised the therapy of several solid tumours [6]. Although PTX continues to be successfully used in therapy, including ovarian, lung, and breast cancers, its use is limited by low solubility, toxicity, and the development of drug resistance. The toxicity of PTX is associated with severe adverse side effects, including hypersensitivity reactions, myelosuppression, bradycardia, hypotension, peripheral neuropathy, myalgias, arthralgias, nausea, diarrhoea, mucositis, and alopecia. Additionally, some adverse effects may not be directly attributed to PTX itself but arise from the use of toxic solvents (most commonly Cremophor EL) required to solubilise this highly hydrophobic drug [7]. These adverse effects may be so persistent or even life-threatening that they can cause delays in treatment continuation, dose reductions in subsequent cycles, or even treatment discontinuation, which may have serious implications for the patient’s survival chances.

A potential alternative treatment approach involves medications that, when administered alongside PTX, could enhance its effectiveness without substantially increasing its harmful effects. These compounds might enable the use of reduced PTX dosages in cancer treatment, potentially limiting the development of drug resistance and decreasing side effects unrelated to microtubule activity [8]. PTX and docetaxel are usually combined with radiotherapy [9] and other chemotherapeutics, such as cisplatin [10], carboplatin and trastuzumab [11], doxorubicin [12], or gemcitabine [13,14]. Recent studies also include combining PTX with immunotherapy and hormone therapy in the treatment of triple-negative breast cancer [15,16]. It is well documented that combining chemotherapy with surgery and radiotherapy (adjuvant or neoadjuvant therapy) or using multidrug chemotherapy is more effective than monotherapy [17]. A well-established strategy for improving treatment efficacy is the combined use of taxanes and tubulin polymerisation inhibitors, two classes of drugs with opposite mechanisms of action against microtubules. The most frequently tested combination is the co-administration of PTX and vinorelbine, and the results of in vitro, in vivo, and clinical trials have shown favourable activity and toxicity profiles [18].

**Figure 1:**
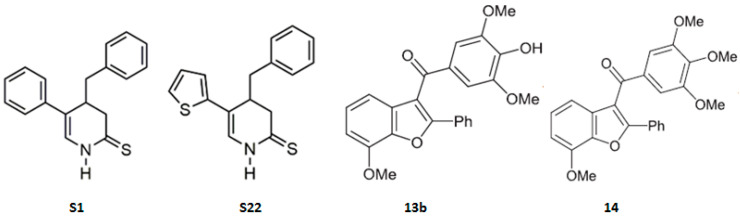
Structure of pyridine derivatives (S1, S22) [20,21] and benzofuran derivatives (13b, 14) [22,23].

The novelty of the solution proposed in this paper lies in the combination of PTX and new tubulin polymerisation inhibitors at doses that are safe but clinically irrelevant when the compounds are used alone. The research hypothesis is based on previous studies, which showed that the carbazole Carba1, a colchicine binding-site inhibitor (CBSI), that exhibits minimal cytotoxicity (GI_50_ = 21.8 µM) and moderate in vitro anti-tubulin activity (IC_50_ = 6.9 µM), induced subtle alterations in microtubule dynamics at the growing extremity of microtubules. These alterations led to the formation of lattice regions that enhanced the accumulation of PTX inside the microtubules, accounting for the observed synergism of action [19]. The aim of the current study was to evaluate the therapeutic efficacy of combining low, non-toxic doses of PTX with previously described pyridine derivatives (S1, S22) [20,21] and benzofuran derivatives (13b, 14) [22,23] (Figure 1, Table 1), which show promising anticancer properties related to the inhibition of microtubule polymerisation. All tested derivatives used alone disrupted mitotic spindles by binding to the colchicine site on tubulin, leading to cell cycle arrest and apoptosis. The aim of this study was to determine the effect of combining compounds on the viability of cancer cells, cell cycle distribution, apoptosis, and morphology of mitotic spindles. The obtained results will enable us to determine if combining use of two classes of drugs with opposing mechanisms of action against microtubules can help decrease the dosage of the widely used PTX in order to limit the side effects of chemotherapy, while maintaining or even increasing its effectiveness. It is believed that combining substances that enhance or maintain microtubule formation with those that induce subtle modifications of microtubule dynamics could amplify the pharmacological action of taxanes.

## 2. Materials and Methods

### 2.1. Tested Compounds

Pyridine (S1, S22) and benzofuran (13b, 14) derivatives were synthesised based on previously described methods and provided in powder form [20,22]. The compounds were then dissolved in dimethyl sulfoxide (DMSO) (Sigma-Aldrich Merck Group, St. Louis, MO, USA). PTX was purchased from Cytoskeleton (Denver, CO, USA), also being dissolved in DMSO, and stored at −20 °C as a 2 mM stock solution.

### 2.2. Cell Culture

The HeLa and HeLa Kyoto cell lines were provided courtesy of Prof. Laurence Lafanechère from the Institute for Advanced Biosciences in Grenoble, France. Cell cultures were maintained in a humidified incubator at 37 °C with 5% CO_2_ in an RPMI medium supplemented with 10% heat-inactivated FBS (EURx, Gdansk, Poland), 2 mM L-glutamine (Sigma-Aldrich Merck Group), and a penicillin–streptomycin solution (Sigma-Aldrich Merck Group). The cell lines were regularly tested for mycoplasma contamination using the MycoAlert^®^ Mycoplasma Detection Kit (Lonza, Rockland, ME, USA).

### 2.3. Evaluation of Cell Viability with PrestoBlue Assay

HeLa cells were seeded in 96-well black microplates at a density of 2.5 × 10^3^ cells per well and allowed to grow under standard conditions for 24 h before being treated for 72 h with a medium containing DMSO (0.2%), PTX, 13b, 14, S1, S22, or combinations of PTX + 13b/14/S1/S22 across a wide concentration range. The range of tested concentrations varied between the compounds, as follows, for PTX: 0.5, 1, 2, 2.5, 5, and 7.5 nM; for 13b: 0.1, 0.5, 1, 2, and 5 µM; for 14: 0.01, 0.05, 0.1, 0.5, and 1 µM; and for S1/S22: 0.5, 1, 2, 5, and 10 µM. The above differences resulted from the different activity of the compounds, and the concentration range was selected so that the IC50 values were in the middle of the scale. In each well, the final concentration of DMSO did not exceed 0.2%. After 72 h, 10 µL of a PrestoBlue reagent (Thermo Fisher Scientific, Waltham, MA, USA) was added to the cells and incubated for 30 min. Fluorescence was then measured (excitation: 540 nm, emission: 590 nm) using a spectrophotometric microplate reader (Infinite 200 Pro; Tecan, Männedorf, Switzerland). Cell viability was determined using the following formula: (Atest/Acontrol) × 100%. The measurements were obtained from three independent experiments.

### 2.4. Analysis of Drug Interactions

The optimal concentrations for drug combinations (PTX + 13b/14/S1/S22) were determined using the Q-value method [24]. First, the inhibition rate was calculated using the following formula: (100 − viability)/100. The Q-value was then determined using the following formula: Q = E(A + B)/[(EA + EB) − (EA × EB)]. Here, EA represents the inhibition rate for drug A, EB is the inhibition rate for drug B, and E(A + B) is the inhibition rate for the combined treatment. A Q-value between 0.85 and 1.15 suggests additive effects, a Q-value greater than 1.15 indicates a synergistic effect, and a Q-value less than 0.85 signifies an antagonistic interaction.

### 2.5. Apoptosis Assay

HeLa cells were plated in 24-well plates at a density of 1.5 × 10^4^ cells per well. After 24 h of incubation under standard conditions, the cell culture medium was removed, and a fresh medium containing the optimal concentrations for combinations—13b (1 µM) + PTX (0.001 µM), 14 (0.1 µM) + PTX (0.001 µM), S1 (2 µM) + PTX (0.001 µM), and S22 (2 µM) + PTX (0.001 µM)—and the compounds alone at the same concentrations was added. The cells were incubated for an additional 72 h. Cells grown in the medium with 0.2% DMSO served as the control. Post treatment, the cells were washed with phosphate-buffered saline (PBS) (both the cell medium and PBS were collected) and harvested via trypsinisation. After centrifugation, the supernatant was discarded, and the cell pellet was resuspended in a fresh medium. An equal volume of a Muse Annexin V & Dead Cell Reagent (Luminex Corporation, Austin, TX, USA) was added and mixed thoroughly by pipetting, and the cells were stained for 20 min at room temperature in the dark. The samples were then analysed using the Muse Cell Analyzer (Luminex Corporation). This assay utilises Annexin V-PE to detect phosphatidylserine on the outer membrane of apoptotic cells and a dead cell marker (7-AAD) as an indicator of cell membrane integrity. The combined staining with these two dyes allows for the identification of four cell populations: viable cells (Annexin V-PE− and 7-AAD−), early apoptotic cells (Annexin V-PE+ and 7-AAD−), late apoptotic or dead cells (Annexin V-PE+ and 7-AAD+), and mostly nuclear debris (Annexin V-PE− and 7-AAD+). The results were obtained from at least three independent experiments.

### 2.6. Cell Cycle

HeLa cells were plated in six-well plates at a density of 7.5 × 10^4^ cells per well and incubated for 24 h under standard conditions. After this period, the medium was replaced with a fresh medium containing the optimal concentrations for combinations, 13b (1 µM) + PTX (0.001 µM), 14 (0.1 µM) + PTX (0.001 µM), S1 (2 µM) + PTX (0.001 µM), and S22 (2 µM) + PTX (0.001 µM), and the compounds alone at the same concentrations. The cells were then incubated for an additional 5 and 24 h. Control cells were maintained in a medium with 0.2% DMSO. Following treatment, the cells were washed with PBS and harvested using trypsin. The cell pellet was then washed with PBS and fixed in 70% cold ethanol (Chempur, Piekary Śląskie, Poland) at −20 °C for at least 3 h. Before the analysis, the ethanol-fixed cells were centrifuged, washed with PBS, and stained with a Muse Cell Cycle Reagent (Luminex Corporation), which contains propidium iodide (PI) and RNAse, for 30 min at room temperature in the dark. The Muse Cell Analyzer (Luminex Corporation) was used to measure DNA content based on PI staining, allowing for the determination of the percentage of cells in each phase of the cell cycle. The results were obtained from at least three independent experiments.

### 2.7. Live-Cell Imaging

For live-cell imaging, HeLa Kyoto cells expressing EGFP-alpha-tubulin were seeded in 96-well black plates at a density of 2.5 × 10^3^ cells per well and allowed to grow under standard conditions for 24 h. The medium was then replaced with a fresh medium containing the optimal concentrations for combination S1 (2 µM) + PTX (0.001 µM), the compounds alone at the same concentrations, and additionally PTX at 0.005 µM, and 0.2% DMSO as the control. The cell culture plate was immediately placed in the imaging chamber of an Agilent BioTek Lionheart FX automated microscope (Agilent Technologies, Inc., Santa Clara, CA, USA) equipped with a Plan Fluorite 20×/0.45 objective (Olympus, Nishi-Shinjuku, Tokyo, Japan) and an FLIR Blackfly BFLY-U3-23S6M camera (Teledyne FLIR, Wilsonville, OR, USA). The temperature in the imaging chamber was set to 37 °C, and the CO_2_ level was maintained at 5%. Images were captured every 5 min for 24 h using the appropriate settings for EGFP (469/35 nm excitation and 525/39 nm emission filters, 14 ms integration time, 24 gain, 10 illumination). All quantitative data were acquired from two independent experiments.

### 2.8. Statistical Analysis

Results are presented as the mean ± standard error of the mean (SEM). The statistical analysis was performed using Statistica 12 software (StatSoft Inc., Tulsa, Oklahoma, USA). Statistical differences between groups were evaluated using an analysis of variance (ANOVA), followed by Tukey’s multiple comparisons test where appropriate. A *p*-value <0.05 was considered statistically significant.

## 3. Results

### 3.1. Selection of Optimal Synergistic Concentrations Through Cell Viability Screening

**Figure 2:**
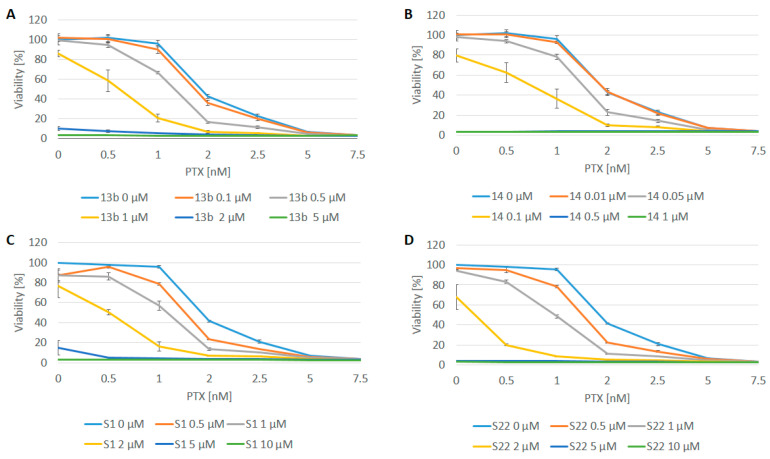
Dose–effect curve. (**A**) Effect of PTX in combination with compound 13b. (**B**) Effect of PTX in combination with compound 14. (**C**) Effect of PTX in combination with compound S1. (**D**) Effect of PTX in combination with compound S22 on viability of HeLa cells determined by PrestoBlue assay after 72 h of treatment. Data are presented as mean ± SEM of three independent experiments. Note that effect of different doses alone on cell viability can be seen at x-coordinate = 0, corresponding to 0 µM of PTX.

**Figure 3:**
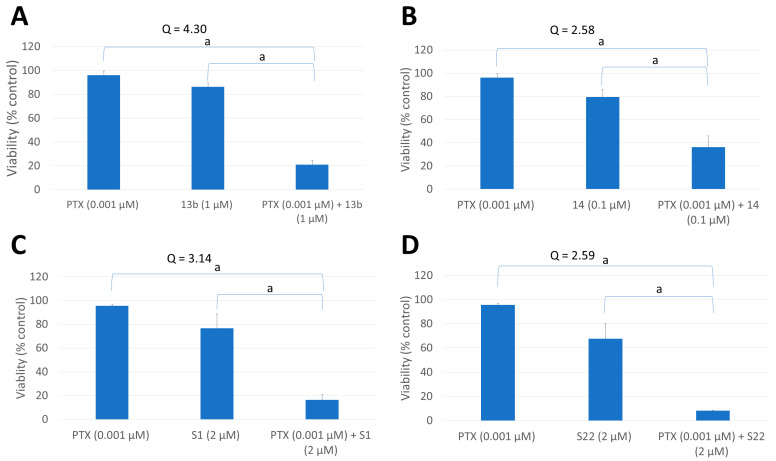
The effect of selected doses of PTX and compounds. (**A**) The effect of PTX in combination with compound 13b. (**B**) The effect of PTX in combination with compound 14. (**C**) The effect of PTX in combination with compound S1. (**D**) The effect of PTX in combination with compound S22 on the viability of HeLa cells determined by the PrestoBlue assay after 72 h of treatment. Data are presented as the mean ± SEM of three independent experiments. The graphs also show Q-values. According to the method described by Yao et al. [24], Q-values exceeding 1.15 indicate a synergistic interaction between the two compounds. Statistical significance was determined by ANOVA followed by Tukey’s post hoc test (^a^*p* < 0.05).

The aim of the first stage of this research was to evaluate the effect of pyridine and benzofuran derivatives (administered separately and in combination with PTX) on the viability of cancer cells and to determine the optimal concentrations for the drug combinations. The results, obtained using the PrestoBlue method (which measured the viability of HeLa cells after 72 h of incubation with various concentrations of compounds 13b, 14, S1, and S22) are shown in Figure 2. As expected, the viability of HeLa cells decreased in a dose-dependent manner upon treatment with the drugs, whether used alone or in combination. The IC_50_ of PTX was 1.83 ± 0.03 nM. Minimal concentrations of PTX (0.5 and 1 nM) had a negligible effect on cell viability, which ranged between 95.58% and 101.90%. The compounds tested independently exhibited moderate toxicity, with IC_50_ values of 1.33 ± 0.11 µM, 0.15 ± 0.04 µM, 3.25 ± 1.19 µM, and 2.37 ± 0.49 µM for 13b, 14, S1, and S22, respectively. In combination, the most pronounced effects were observed when various concentrations of PTX were combined with 1 μM of 13b, 0.1 μM of 14, and 2 μM of S1 and S22 (yellow lines in Figure 2). Moreover, the introduction of these concentrations led to a reduction in the IC_50_ values of PTX, specifically by 3.3, 2.6, 3.8, and 8.8 times in the presence of 13b, 14, S1, and S22, respectively. Based on the results and the Q-values (the combination index determined using the method described by Yao et al. [24]) (Table S1), the optimal concentrations of the combined compounds were determined. These concentrations were those at which cell viability did not change (or changed only slightly when the compounds were used alone) but decreased markedly when the compounds were used in combination. As shown in Figure 3, for all compounds at the selected concentrations (i.e., 1 μM, 0.1 μM, 2 μM, and 2 µM for 13b, 14, S1, and S22, respectively), their combination with a non-toxic dose of PTX (1 nM) significantly reduced cell viability compared with the compounds used alone and exhibited clear synergism (Q-values of >1.15).

### 3.2. Effect of Compound Combination on Cell Apoptosis

**Figure 4:**
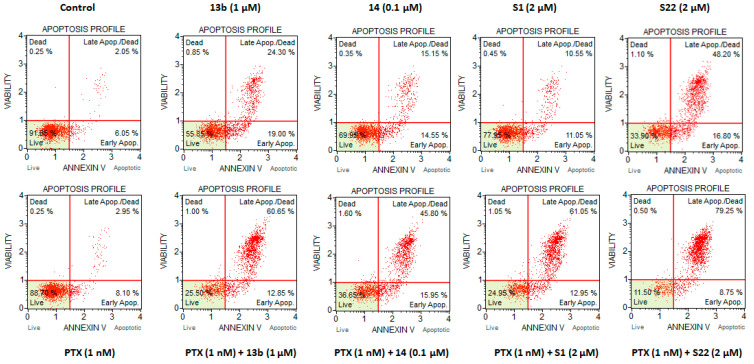
Representative scatter diagrams of HeLa cells exposed to the indicated concentrations of compounds 13b, 14, S1, and S22 alone and in combination with PTX, after 72 h of treatment. Cells were subsequently stained with Annexin V-PE and 7-AAD and analysed using the Muse Cell Analyzer.

**Figure 5:**
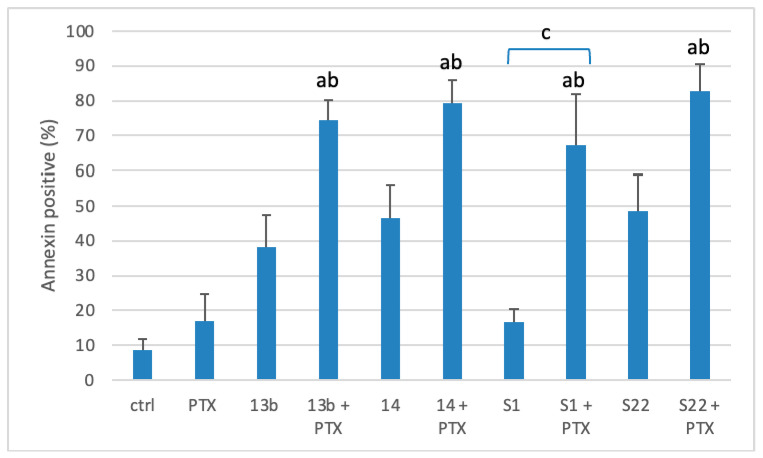
The effect of selected concentrations of PTX (1 nM) and compounds. The figure shows the effect of PTX (1 nM) and compounds 13b (1 µM), 14 (0.1 µM), S1 (2 µM), and S22 (2 µM), alone and in combination, on the induction of apoptosis in the HeLa cell line after 72 h of treatment. Cells were subsequently stained with Annexin V-PE and 7-AAD and analysed using a Muse Cell Analyzer. Data are expressed as the mean ± SEM from at least three independent experiments. Statistical significance was determined by ANOVA followed by Tukey’s post hoc test (^a^ *p* < 0.05 vs. control, ^b^ *p* < 0.05 vs. PTX, ^c^ *p* < 0.05, compound alone, vs. in combination with PTX).

As apoptosis is a known mechanism of cytotoxicity for both PTX [7] and the tested pyridine [20] and benzofuran [22] derivatives, the next step involved directly detecting apoptotic cells using an apoptotic marker and quantifying them by flow cytometry. Representative experiments, showing cell populations divided into quadrants in dot plots based on Annexin V-PE/7-AAD staining, are illustrated in Figure 4. As shown in Figure 5, which displays the population of annexin-positive cells, HeLa cells treated with a combination of PTX (1 nM) and 13b (1 µM), 14 (0.1 µM), S1 (2 µM), and S22 (2 µM) exhibited a considerable increase in the apoptotic cell population to 74.4%, 79.43%, 67.38%, and 82.8%, respectively, compared with the control (8.75%) and PTX alone (16.95%). Moreover, the percentage of apoptotic cells observed for the combinations was higher than the sum of percentages measured for PTX and each compound when applied individually (55.28% for PTX + 13b, 63.5% for PTX + 14, 33.5% for PTX + S1, and 65.6% for PTX + S22), highlighting their synergistic action. Although the differences between the compounds used alone and their combinations with PTX were only significant for S1, all combinations were significantly different from PTX alone. For S1, the percentage of apoptotic cells increased from 16.55% when used alone to 67.38% when combined with PTX. The percentage of apoptotic cells for compounds used individually, including PTX, was higher than suggested by the PrestoBlue results, underscoring the sensitivity of the apoptosis assay.

### 3.3. Effect of Compound Combination on Cell Cycle

**Figure 6:**
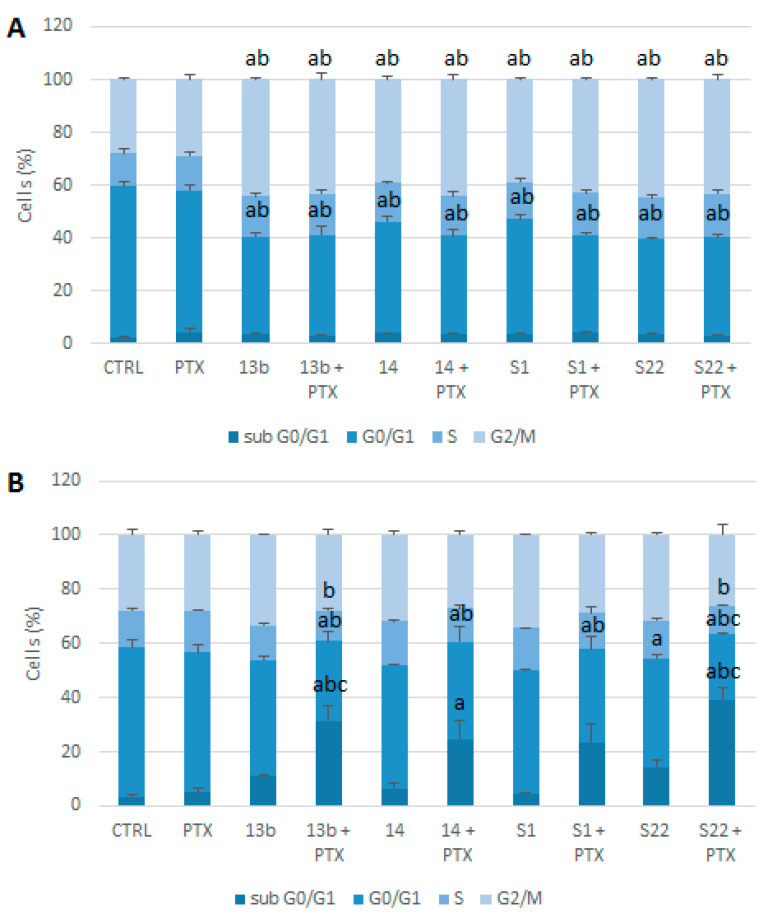
(**A**) The effect of PTX (1 nM) and compounds 13b (1 µM), 14 (0.1 µM), S1 (2 µM), and S22 (2 µM), alone and in combination, on the cell cycle distribution in the HeLa cell line after 5 h of treatment. (**B**) The effect after 24 h of treatment. Cells were fixed with 70% ethanol, stained with propidium iodide, and analysed using a Muse Cell Analyzer. Data are expressed as the mean ± SEM from three independent experiments. Statistical significance was determined by ANOVA followed by Tukey’s post hoc test (^a^ *p* < 0.05 vs. control, ^b^ *p* < 0.05 vs. PTX, ^c^ *p* < 0.05, compound alone, vs. in combination with PTX).

**Figure 7:**
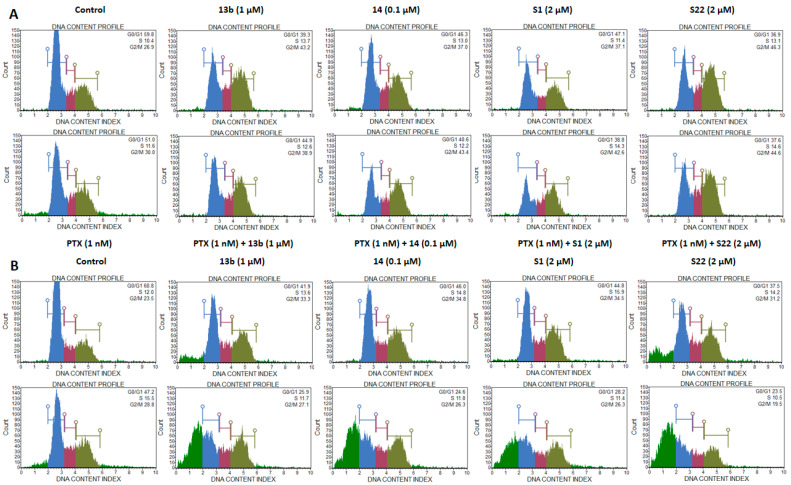
(**A**) Representative individual experiment showing cell cycle phase distribution of HeLa cells exposed to indicated concentrations of compounds 13b, 14, S1, and S22 alone and in combination with PTX, after 5 h of treatment and (**B**) after 72 h of treatment. Cells were fixed with 70% ethanol, stained with propidium iodide, and analysed using Muse Cell Analyzer.

The known mechanisms of the action of PTX and the tested pyridine and benzofuran derivatives led us to assume that the observed toxicity of the combinations may result from arresting cells in mitosis. The flow cytometry analysis using propidium iodide staining revealed that exposing cells to the compounds alone or in combination with PTX for 5 h induced G2/M phase cell cycle arrest and a subsequent reduction in the number of cells in the G0/G1 phase (Figure 6A). Notably, the concentration of PTX used did not alter the cell cycle distribution compared with the control, even when the treatment duration was extended to 24 h (Figure 6B). Prolonged exposure led to a reduction in the number of cells blocked in the G2/M phase and a significant increase in the number of aneuploid cells, evident as a sub-G0/G1 population. This increase was particularly pronounced in the combinations of 13b, 14, and S22 with PTX. The 13b/PTX and S22/PTX combinations significantly elevated the apoptotic cell population (31.42% and 39.31%, respectively), even compared with the compounds used individually (10.98% for 13b and 14.44% for S22). All combinations showed a substantial decrease in the G0/G1 phase cell population after 24 h of incubation compared to both the control and PTX alone. Representative individual experiments are shown in Figure 7.

### 3.4. Live-Cell Imaging

**Figure 8:**
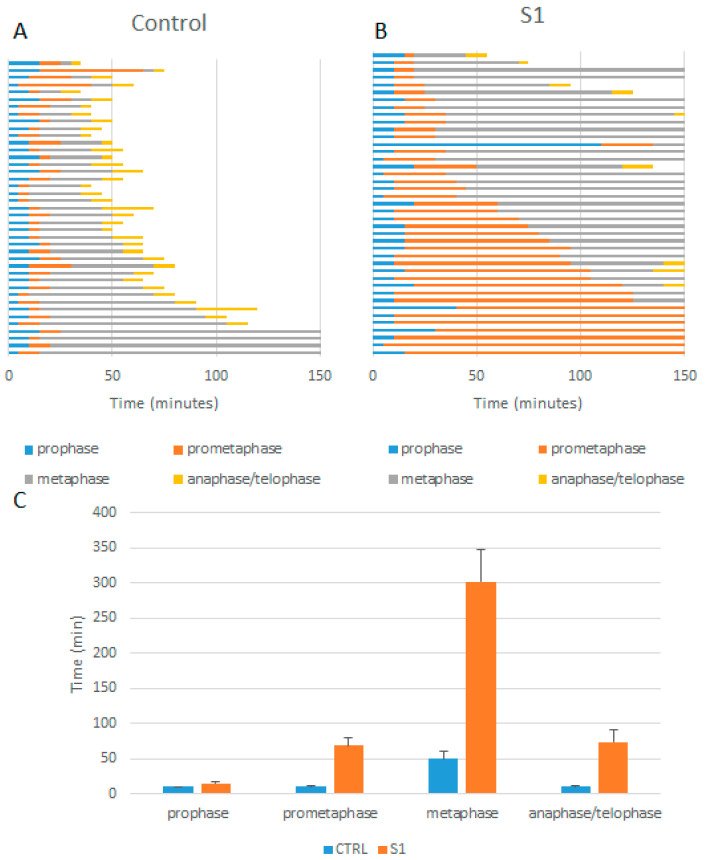
The analysis of the duration of mitosis in HeLa Kyoto cells. (**A**) HeLa Kyoto cells treated with 0.2% DMSO (control). (**B**) HeLa Kyoto cells treated with 2 µM S1. The duration of prophase [from nuclear envelope breakdown (NEBD); blue], prometaphase (from the appearance of mitotic spindles; orange), metaphase (from the appearance of the metaphase plate; grey), and anaphase/telophase (from chromosome separation to cytokinesis; yellow) was analysed from Videos S1 and S2 in the Supplementary Materials. The data represent 41 cells for each treatment from two independent experiments.

**Figure 9:**
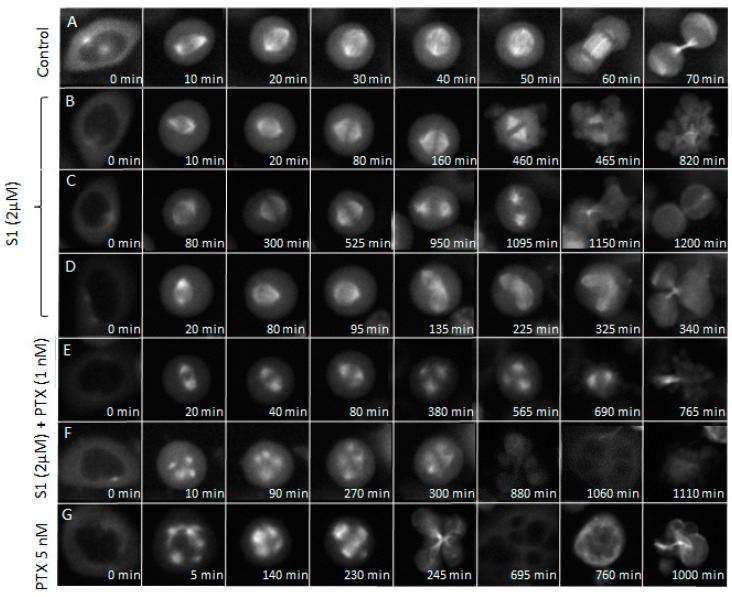
Representative images, selected from Videos 1–5 in the Supplementary Materials, of HeLa Kyoto cells treated with 0.2% DMSO (control) (**A**), S1 (2 µM) (**B**–**D**), S1 (2 µM) + PTX (1 nM) (**E**,**F**), PTX (5 nM) (**G**).

Because S1 was the compound that had the most synergistic effect with PTX, we analysed its effect on mitosis, both alone and in combination with PTX, using live-cell imaging of HeLa cells expressing EGFP-alpha-tubulin (HeLa Kyoto cells) (Supplementary Materials, Videos S1–S5). The careful analysis of the videos (Table 2) revealed that the percentage of aberrant mitosis increased from 12.20% (control, 0.2% DMSO) and 23.08% (1 nM PTX) to 64.91% (2 µM S1) and 80.65% (1 nM PTX and 2 µM S1). Moreover, no normal mitosis was observed with the compound combination. Additionally, we observed that in the case of compound S1 alone and its combination with PTX, a similar proportion of mitotic cells (19.3% and 19.35%, respectively) eventually died during delayed mitosis. We then calculated from the videos the time spent in each mitotic phase (Figure 8). We found that compound S1, compared with the control, significantly extended the duration of mitosis, particularly during prometaphase and metaphase. As shown in Figure 9B, this mitotic delay was often followed by apoptosis. Other scenarios were also observed; for instance, as shown in Figure 9C, extended mitosis (in this case, anaphase) could result in the formation of two apparently normal daughter cells, whereas Figure 9D illustrates aberrant mitosis, in which two metaphase plates formed, ultimately leading to the generation of four cells.

When S1 was applied in combination with 1 nM PTX, a characteristic feature was the appearance of multipolar mitotic spindles, either emerging after an initially apparently normal metaphase (Figure 9E) or immediately after the onset of mitosis (Figure 9F). These spindles resulted either in apoptosis (Figure 9E) or in the formation of multinucleated cells (Figure 9F). Such multipolar spindles resembled those observed in cells exposed to high toxic concentrations of PTX (5 nM), as shown in Figure 9G. Notably, the multinucleated cells continued to undergo abnormal division and eventually entered apoptosis, as shown in Figure 9G.

## 4. Discussion

A cancer treatment regimen that combines two or more therapeutic agents is currently considered a standard option in chemotherapy. Such a therapeutic approach, by simultaneously targeting multiple critical molecular pathways, increases the effectiveness of therapy and allows for reduced drug doses, thereby decreasing side effects. Moreover, compared with monotherapy, combination therapy effectively prevents drug resistance [25]. PTX is a widely used and effective chemotherapy drug, employed either alone or in combination with other anticancer agents depending on the type or stage of cancer [1]. To our knowledge, the combined use of taxanes and Vinca alkaloids—two classes of drugs with opposite mechanisms of action against microtubules, the efficacy of which has been well documented in the literature—is not currently employed in clinical practice.

In the present study, we investigated the ability of low doses of previously described pyridine (S1, S22) [20,21] and benzofuran [22]-derived tubulin inhibitors to potentiate the anticancer effects of low doses of PTX. We used the well-established cervical cancer cell line HeLa, which has confirmed sensitivity to PTX [26]. Based on the results of the PrestoBlue assay and the combination index (Q-value) calculation, we determined the optimal concentrations of each compound to act in synergy with 1 nM PTX. Clear synergism (Q-value > 1.15) was observed for 1 μM of 13b, 0.1 μM of 14, 2 μM of S1, and 2 µM of S22. Moreover, at these concentrations, the compounds exhibited minimal cytotoxic activity when applied alone. The results of the present study are consistent with previous studies on HeLa cells, indicating that 1 nM PTX acted synergistically with the tubulin polymerisation inhibitor Carba1 [19] and the DNA flap endonuclease 1 (FEN1) inhibitor [27]. Much higher concentrations of PTX were required for its synergistic interaction with natural compounds. For example, the highest synergistic rates in CL1–5 lung cancer cells were observed with a combination of 12.5 μM 5-demethylnobiletin (5-DMN), a compound isolated from orange peel, and 10 nM PTX [28]. The potentiating effect of co-treatment with PTX and natural compounds was also reported by Asnaashari et al. [29], who demonstrated that the anticancer activity of PTX increased when combined with numerous flavonoids [29]. The need for higher concentrations of PTX with natural compounds results from a different mechanism of action responsible for the synergism. Most studies have demonstrated that the activation of a caspase-dependent apoptotic pathway is a major mechanism making cancer cells more susceptible to PTX-triggered apoptosis.

Our study revealed that HeLa cells treated with a combination of PTX (1 nM) and 13b, 14, S1, or S22 at the selected concentrations exhibited a considerable increase in the apoptotic cell population compared to the control and PTX alone. Furthermore, the level of apoptosis induced by the combination of PTX and S1 was significantly higher than that induced by S1 alone.

The most common cellular effect of compounds targeting microtubules is the interruption of cell division during mitosis. Drugs that restrict the dynamic nature of spindle microtubules hinder their ability to attach to chromosomes during metaphase and the entire mitotic process. This disruption results in a halt to mitotic progression and ultimately leads to cell death triggered by checkpoint mechanisms [1]. As previous studies have shown, both pyridine [20] and benzofuran [22] tubulin polymerisation inhibitors arrest cancer cells in mitosis. After 6 h of the incubation of melanoma A375 cells with 10 µM S1 and S22, nearly half of the cell population were arrested in the G2/M phase. However, prolonged exposure (48 h) led to a reduction in the number of cells blocked in the G2/M phase and a significant increase in the number of hypodiploid DNA (sub-G0/G1) and/or polyploid DNA (population post-G2/M phase) [20]. A similar effect was observed by Kwiecień et al. [22] after 7 h of the incubation of A375 cells with compounds 13b (100 µM) and 14 (50 µM).

In the present study, the flow cytometric analysis showed that exposing cells to low doses of the compounds, either alone or in combination with PTX, caused G2/M phase cell cycle arrest. It should be emphasised that PTX at 1 nM did not alter the cell cycle distribution compared with the control. Subsequently, the apoptotic sub-G1 phase increased markedly in the combinations of 13b, 14, and S22 with PTX, suggesting the occurrence of sequential events of cell cycle arrest followed by apoptosis. Arresting cells in the G2/M phase is indicated as one of the mechanisms underlying the synergistic interaction of PTX with natural compounds [29]. Sánchez-Carranza et al. [30] explained this phenomenon through the ability of gallic acid to potentiate PTX-induced G2/M phase arrest. Their research on a drug-resistant cell line revealed that gallic acid triggers an overproduction of reactive oxygen species, which in turn leads to proliferation inhibition and G2/M phase arrest. Additionally, these reactive oxygen species interfere with the PTX-induced activation of extracellular signal-regulated kinases (ERKs).

Cell arrest in mitosis was confirmed by a further videomicroscopic analysis. The phase-specific mechanism of action of MTAs can lead to several outcomes, including cell death during mitosis, mitotic exit, programmed cell death, or aneuploidy [31]. In the present work, mitotic arrest led to apoptosis or, in the case of the compound combination, the formation of multinucleated cells. It should be emphasised that the effect observed during the videomicroscopy analysis using S1 and PTX concurrently resembles the effects of PTX at a higher dose. It is well known that the impact of PTX on microtubules varies with its concentration: high doses cause a complete halt in mitosis [32], while low concentrations result in improper chromosome distribution [33]. Our observations align with the findings of Peronne et al. [19], who noted that Carba1 enhances the effect of a low dose of PTX (1 nM), producing a response similar to 5 nM PTX. Given that low doses of PTX alone did not significantly disturb the cell cycle or division spindle organisation in HeLa cells, we conclude that S1, similar to Carba1, sensitised cancer cells to PTX. The synergistic effect of our compounds with PTX at the microtubule level further highlights the absence of this effect at higher doses.

The ability of microtubule-targeting agents to bind to many distinct locations on tubulin dimers suggests that the use of several of these drugs in a clinic could potentially improve treatment efficacy and minimise adverse effects [18]. As shown by current research and available literature data, the key factor for the synergistic interaction between the proposed combination of compounds is the opposite anti-tubulin effect and the use of low doses of the drugs. While research has demonstrated that the covalent binding at the taxane site can influence the structure of the colchicine site [34], the opposite effect has not been observed or reported yet. As suggested by Peronne et al., it is unlikely that the simultaneous binding of PTX and the depolymerising compound on one tubulin dimer is responsible for the observed synergistic effect. However, Peronne et al. [19] and Rai et al. [35] demonstrated that MT-depolymerising compounds (such as vinblastine or Carba1) at non-saturating doses induce changes in the dynamics of microtubules at their tips, favouring transitions to catastrophes (shifts from growth to depolymerisation). It has been shown with fluorescent PTX that the latter accumulates preferentially during these transitions, resulting in a higher amount of PTX being bound. The findings indicate that enhanced taxane integration near a microtubule’s growing end results in the stabilisation of a stretch of the microtubule lattice. This phenomenon explains why, at the cellular level, an effect similar to that of higher PTX concentrations is observed. Peronne et al. [19] also suggested that the synergistic interaction between PTX and Carba1 might involve mechanisms independent of MT dynamics, proposing that Carba1 increases PTX accumulation in cells by inhibiting efflux pumps. This intriguing suggestion could also apply to our compounds but requires further investigation.

Moreover, recent in vivo research, which allows for the examination of tumour immune microenvironment reactions to taxanes, has revealed a mechanism of action independent of mitosis. Research by Vennin et al. [36] demonstrated that taxanes stimulate T cells to release cytotoxic extracellular vesicles, which, in turn, lead to tumour cell death. In light of these findings, it would be interesting to investigate the effect of the PTX combination on dendritic cells, which play a key role in stimulating the immune response.

Multipolar metaphase and multinucleation allowed us to define the mitotic defect observed under the influence of S1 + PTX as a mitotic catastrophe. It is worth emphasising, however, that a mitotic catastrophe does not always lead to apoptosis. Cell death resulting from a mitotic catastrophe is referred to as mitotic death and most often occurs through the activation of the intrinsic apoptosis pathway, although necrosis and autophagy are also possible [37,38] Moreover, inducing apoptosis in cancer cells typically requires high concentrations of chemotherapeutic agents. However, it has been shown that a mitotic catastrophe can be triggered by significantly lower doses of therapeutic agents, including taxanes and Vinca alkaloids. Increasingly, the induction of a mitotic catastrophe is being recognised as a promising strategy for cancer prevention and treatment [39].

## 5. Conclusion

The high rates of cancer-related illness and death, coupled with the limited efficacy and adverse effects of current treatments, have driven the search for new therapeutic approaches. One such approach may involve combining traditional chemotherapy drugs like PTX with new mitosis inhibitors. In this study, the combined effects of a low, non-toxic dose of PTX and four tubulin polymerisation inhibitors were explored to reduce the currently used doses of PTX and to limit chemotherapy-related adverse effects. The results revealed that compounds 13b (1 µM), 14 (0.1 µM), S1 (2 µM), and S22 (2 µM) worked synergistically with 1 nM PTX to diminish HeLa cell viability in vitro. Moreover, the co-administration of PTX with mentioned pyridine and benzofuran derivatives was found to enhance the proapoptotic and antimitotic impacts of low-dose PTX, which was otherwise ineffective when used alone. Additionally, the spindle morphology alterations observed under the combined treatment resembled those characteristic of higher, more toxic doses of PTX. Thus, the combination of low doses of MTAs with opposing mechanisms of action could potentially improve the efficacy and safety of cytostatic drugs that stabilise MTs and are already available on the pharmaceutical market, offering a promising new strategy in cancer treatment. However, further investigation is required to elucidate the molecular mechanisms driving this synergistic effect. Future in vivo studies will also require the selection of the optimal drug formulation for the co-administration of both components and maintaining the bioavailability of PTX.

## Figures and Tables

**Table 1:** Anticancer activity of previously described pyridine derivatives (S1, S22) [20,21] and benzofuran derivatives (13b, 14) [22,23].

	S1	S22	13b	14
IC_50_ (A375, WST-1 assay)	4.3 µM	1.7 µM	0.9 µM	0.09 µM
IC_50_ (tubulin polymerisation assay in cell-free conditions)	10.8 µM	26.8 µM	<3 µM	<3 µM

**Table 2:** The quantification of the fate of HeLa Kyoto cells treated with 0.2% DMSO (control), PTX (1 nM), and S1 (2 µM), alone and in combination, observed over 24 h using time-lapse videomicroscopy; *n* represents the number of mitotic cells analysed from two independent experiments.

	Control (%)	PTX (%)	S1 (%)	S1 + PTX (%)
**Normal mitosis**	87.80	75.38	15.79	0.00
**Abnormal mitosis**	12.20	23.08	64.91	80.65
**Arrested mitosis followed by apoptosis**	0.00	1.54	19.30	19.35
	*n* = 41	*n* = 65	*n* = 57	*n* = 62

## Data Availability

The data presented in this study are available on request from the corresponding author.

## Supplementary Materials

The following supporting information can be downloaded at: https://www.mdpi.com/article/10.3390/pharmaceutics17020223/s1, Table S1: Q-values (combination index) for each combination. Data are presented as the mean ± SEM from three independent experiments. A Q-value of 0.85–1.15 indicates the sum of the effects, a Q-value of >1.15 indicates a synergistic effect, and a Q-value of <0.85 indicates an antagonistic effect of the combined drugs. The selected combinations are highlighted in blue. Video S1: control (0.2% DMSO), Video S2: S1 (2 µM), Video S3: PTX (1 nM), Video S4: S1 (2 µM)_PTX (1 nM), Video S5: PTX (5 nM).

## Abbreviations

The following abbreviations are used in this manuscript:

5-DMN	5-Demethylnobiletin
ANOVA	Analysis of Variance
CBSI	Colchicine Binding-Site Inhibitor
DMSO	Dimethyl Sulfoxide
EGFP	Enhanced Green Fluorescent Protein
ERK	Extracellular Signal-Regulated Kinase
FBS	Foetal Bovine Serum
FEN1	Flap Endonuclease 1
MT	Microtubule
MTA	Microtubule-Targeting Agent
NEBD	Nuclear Envelope Breakdown
PBS	Phosphate-Buffered Saline
PI	Propidium Iodide
PTX	Paclitaxel
SAC	Spindle Assembly Checkpoint
SEM	Standard Error of the Mean
