# Supplementary material for: The Co-Administration of Paclitaxel with Novel Pyridine and Benzofuran Derivatives that Inhibit Tubulin Polymerisation: A Promising Anticancer Strategy
Source: Pharmaceutics. 2025 Feb 9;17(2):223. doi: 10.3390/pharmaceutics17020223 (PMC11859455; doi:10.3390/pharmaceutics17020223)
Supplement: Supplementary file 1 [file pharmaceutics-17-00223-s001.zip › pharmaceutics-3452260-supplementary.pdf]

Supplementary Materials

Table S1. Q-value (combination index) for each combination. Data are presented as mean ± SEM from three independent experiments. A Q-value of 0.85–1.15 indicates the sum of the effects, a Q-value of >1.15 indicates a synergistic effect, and a Q-value of <0.85 indicates an antagonistic effect of the combined drugs. The selected combinations are highlighted in blue.

Dose PTX (nM)	Dose 13b (nM)	Viability	Q-value	Dose 14 (µM)	Viability	Q-value	Dose S1 (µM)	Viability	Q-value	Dose S22 (µM)	Viability	Q-value
0.5	0.1	100.78 ± 3.75	0.69	0.01	100.47 ± 2.91	0.43	0.50	95.46 ± 0.7	0.31	0.50	94.45 ± 2.64	1.04
0.5	0.5	94.92 ± 2.86	0.93	0.05	94.02 ± 1.67	0.99	1.00	86.17 ± 3.54	0.94	1.00	83.18 ± 1.66	2.20
0.5	1	58.64 ± 11	2.68	0.1	62.48 ± 9.62	1.71	2.00	50.31 ± 2.61	2.00	2.00	20.01 ± 1.25	2.37
0.5	2	7.7 ± 1.29	1.03	0.5	3.54 ± 0.18	1.00	5.00	5.11 ± 0.16	1.11	5.00	3.93 ± 0.14	1.00
0.5	5	3.26 ± 0.27	1.00	1	3.2 ± 0.25	1.00	10.00	2.9 ± 0.17	1.00	10.00	2.88 ± 0.09	1.00
1	0.1	90.28 ± 4.01	1.49	0.01	92.69 ± 1.3	0.92	0.50	78.67 ± 1.61	1.28	0.50	78.1 ± 1.42	2.84
1	0.5	66.79 ± 1.34	3.77	0.05	78.55 ± 2.63	2.30	1.00	56.88 ± 4.71	2.57	1.00	48.58 ± 1.63	5.17
**1**	**1**	**20.94 ± 3.91**	**4.30**	**0.1**	**36.34 ± 9.51**	**2.58**	**2.00**	**16.34 ± 4.67**	**3.14**	**2.00**	**8.23 ± 0.25**	**2.59**
1	2	5.18 ± 0.26	1.05	0.5	3.57 ± 0.27	1.00	5.00	4.25 ± 0.23	1.11	5.00	3.67 ± 0.12	1.00
1	5	3.11 ± 0.23	1.00	1	3.21 ± 0.27	1.00	10.00	2.91 ± 0.16	1.00	10.00	2.84 ± 0.13	1.00
2	0.1	36.18 ± 2.78	1.10	0.01	43.07 ± 3.7	0.97	0.50	23.1 ± 0.55	1.21	0.50	22.3 ± 0.39	1.30
2	0.5	16.64 ± 0.95	1.42	0.05	22.84 ± 2.84	1.30	1.00	13.64 ± 1.46	1.35	1.00	10.93 ± 0.46	1.46
2	1	6.61 ± 1.41	1.48	0.1	9.97 ± 1.44	1.36	2.00	6.97 ± 0.52	1.37	2.00	5.38 ± 0.24	1.32
2	2	4.02 ± 0.2	1.00	0.5	3.69 ± 0.21	0.98	5.00	3.68 ± 0.22	1.03	5.00	3.3 ± 0.13	0.98
2	5	3.08 ± 0.23	0.98	1	3.34 ± 0.21	0.98	10.00	2.86 ± 0.17	0.98	10.00	2.71 ± 0.13	0.99
2.5	0.1	20.14 ± 2.04	1.03	0.01	21.57 ± 1.78	1.01	0.50	13.62 ± 0.17	1.06	0.50	13.22 ± 0.94	1.09
2.5	0.5	11.31 ± 1.25	1.13	0.05	14.22 ± 1.63	1.10	1.00	10.11 ± 0.31	1.10	1.00	8.62 ± 0.19	1.14
2.5	1	5.32 ± 0.84	1.18	0.1	7.98 ± 1.04	1.12	2.00	6.28 ± 0.46	1.12	2.00	4.72 ± 0.1	1.11
2.5	2	3.48 ± 0.16	0.99	0.5	3.7 ± 0.21	0.97	5.00	3.45 ± 0.16	1.00	5.00	3.09 ± 0.1	0.98
2.5	5	2.99 ± 0.2	0.98	1	3.34 ± 0.22	0.97	10.00	2.82 ± 0.15	0.98	10.00	2.61 ± 0.12	0.98
5	0.1	6.41 ± 0.41	1.00	0.01	7.24 ± 0.32	0.99	0.50	5.73 ± 0.08	1.00	0.50	5.69 ± 0.13	1.01
5	0.5	4.86 ± 0.28	1.02	0.05	5.54 ± 0.38	1.01	1.00	4.95 ± 0.04	1.01	1.00	4.55 ± 0.1	1.02
5	1	3.1 ± 0.14	1.03	0.1	3.94 ± 0.25	1.02	2.00	3.96 ± 0.19	1.01	2.00	3.48 ± 0.12	1.01

5	2	2.72 ± 0.14	0.98	0.5	3.73 ± 0.27	0.97	5.00	3 ± 0.13	0.98	5.00	2.75 ± 0.1	0.98
5	5	3.01 ± 0.2	0.97	1	3.48 ± 0.21	0.97	10.00	2.62 ± 0.11	0.98	10.00	2.62 ± 0.09	0.98
7.5	0.1	3.57 ± 0.13	1.00	0.01	3.87 ± 0.11	1.00	0.50	3.41 ± 0.06	1.00	0.50	3.58 ± 0.03	1.00
7.5	0.5	3.07 ± 0.16	1.00	0.05	3.34 ± 0.09	1.00	1.00	3.34 ± 0.05	1.00	1.00	3.21 ± 0.07	1.00
7.5	1	2.7 ± 0.12	1.00	0.1	2.94 ± 0.08	1.00	2.00	3.04 ± 0.08	1.00	2.00	2.91 ± 0.06	1.00
7.5	2	2.61 ± 0.13	0.98	0.5	3.92 ± 0.23	0.96	5.00	2.68 ± 0.09	0.98	5.00	2.62 ± 0.07	0.98
7.5	5	3.16 ± 0.15	0.97	1	3.46 ± 0.22	0.97	10.00	2.53 ± 0.09	0.98	10.00	2.56 ± 0.09	0.98
